# Neuroimaging studies of acupuncture for depressive disorder: a systematic review of published papers from 2014 to 2024

**DOI:** 10.3389/fpsyt.2025.1536660

**Published:** 2025-05-15

**Authors:** Dezhi Lin, Qiang Ren, Yangxu Ou, Longlong Li, Dezhong Peng, Sha Yang

**Affiliations:** ^1^ School of Acupuncture and Tuina, Chengdu University of Traditional Chinese Medicine, Chengdu, China; ^2^ Department of Rheumatology and Immunology, Hospital of Chengdu University of Traditional Chinese Medicine, Chengdu, China

**Keywords:** acupuncture, depressive disorder, neuroimaging, brain network, systematic review

## Abstract

**Background:**

Several neuroimaging studies have confirmed that acupuncture can elicit alterations in brain networks and regions associated with depressive disorder (DD). This review provides an overview of the methodologies and results of neuroimaging investigations into the efficacy of acupuncture in treating DD, with the intention of guiding future research objectives.

**Methods:**

Neuroimaging studies of acupuncture for DD being published between February 2, 2014 and February 2, 2024, were gathered from PubMed, Cochrane Library, EMBASE, Web of Science, China National Knowledge Infrastructure, Chongqing VIP Database, WanFang Database, and Chinese Biomedical Literature Database. The methodological quality of the studies was assessed utilizing the Risk of Bias 2.0 and Risk of Bias in Non-Randomized Studies of Interventions tools. Following a qualitative analysis of the studies, relevant information regarding acupuncture interventions and brain imaging data was extracted.

**Results:**

A total of 26 studies met the inclusion criteria. These studies featured a combined sample size of 1138 participants. All studies employed magnetic resonance imaging. Our findings indicate that acupuncture can affect neural activity in the cingulate gyrus, precuneus, insula, prefrontal lobe, etc. The neuroimaging results of most DD patients were correlated with the Hamilton Rating Scale for Depression scores.

**Conclusions:**

The results of the current study indicate that acupuncture treatment may have a regulatory effect on the abnormal functioning of neural regions and networks in individuals diagnosed with DD. These networks are predominantly localized within various brain regions, including the default mode network, limbic system, emotion regulation and cognitive network, reward network, central executive network, salience network, and sensorimotor network. It is essential to conduct additional high-quality and multimodal neuroimaging research to expand upon these findings and elucidate the mechanisms by which acupuncture impacts patients with DD.

**Systematic Review Registration:**

https://www.crd.york.ac.uk/prospero/, identifier CRD42023400557.

## 1 Introduction

According to the World Health Organization, 300 million individuals have been diagnosed with depressive disorder (DD) (1, 2). Although the general incidence of DD is rising, the increase is particularly severe among those aged 13–18 years (3). The prevalence of DD in this population reportedly ranges between 1.5 and 3, with females surpassing males (4). By 2030, DD is projected to become the leading aggravator of disease-related economic and medical burden to patients, their families, and the communities in which they live (5). DD is characterized by severely diminished motivation, the propensity to anger, limited attention, insomnia or drowsiness, fatigue, self-deprecation, and continual suicidal ideation (6, 7).

At present, antidepressants are still the main clinical treatment for DD (8). Although antidepressants are generally effective, they also have certain limitations, including side effects after long-term use, drug resistance, and recurrence (9, 10). Or even worse, antidepressants do not have a relieving effect and instead exacerbate emotional or physical symptoms in some patients, increasing the risk of comorbidity (11, 12). Therefore, it is necessary to find greener, safer, and more effective alternative treatments to improve efficacy and safety.

An increasing body of evidence indicates that acupuncture, as a highly effective non-pharmacological therapy, can significantly improve both the physical and psychological symptoms of patients with DD (13–15). By stimulating specific acupoints, acupuncture engages in multi-level, multi-target regulation, and has been widely utilized in the treatment of DD (16, 17). However, the precise mechanisms underlying acupuncture’s therapeutic effects on depression remain scientifically unclear. With advances in medical imaging techniques, brain imaging-based neuroimaging technologies have become invaluable tools for exploring the mechanisms of acupuncture in treating depression. Neuroplasticity changes play a crucial role in the pathogenesis of DD (18). Alterations in brain neural circuits are direct contributors to the depressive symptoms observed in DD patients (19). Neuroimaging studies have found that the onset of DD is associated with structural and functional abnormalities in specific brain regions involved in emotions and cognition, including the cingulate cortex, prefrontal cortex, amygdala, hippocampus, insula, and striatum (20–24). In resting-state functional magnetic resonance imaging (fMRI), significant changes in these brain regions and nuclei have been observed in individuals with depression, and acupuncture has been found to influence these alterations (25). These brain regions participate in neural networks such as the default mode network (DMN), limbic system, emotion regulation and cognitive network, reward network, and salience network (SN) (26–28). Notably, alterations within and between the DMN, and SN have been highlighted as key findings in many imaging studies on DD (29). Some research suggests that acupuncture may alleviate DD by enhancing the neuroplasticity of brain circuits (15). Furthermore, studies have confirmed that electroacupuncture can modulate the functional activity of the DMN, sensorimotor network, emotion regulation and cognitive network, limbic system, and visual network, thereby exerting therapeutic effects (30).

While brain imaging has been used to provide visual evidence for the benefits that acupuncture affords patients with DD and has helped to elucidate central regulatory mechanisms underpinning these healthful effects, the studies that have collected this evidence were subject to methodological limitations, including their chosen methods of intervention, brain imaging, and analysis. This systematic review seeks to analyze and assess recent neuroimaging studies on the impact of acupuncture on patients with DD in order to identify specific central targets and brain networks influenced by acupuncture therapy. The findings aim to serve as a valuable resource for further clinical research and practice.

## 2 Methods

### 2.1 Registration

This review was performed in accordance with the standards set forth in the Cochrane Handbook for Systematic Reviews of Interventions and followed the Preferred Reporting Items for Systematic Review guidelines (31, 32). The registered study protocol for this systematic review was published in PROSPERO (registration number: CRD42023400557).

### 2.2 Search strategy

The following eight databases were searched for studies published between 2 February 2014 and 2 February 2024: PubMed, Cochrane Library, EMBASE, Web of Science, China National Knowledge Infrastructure, Chongqing VIP Database, WanFang Database, and Chinese Biomedical Literature Database. We restricted our search to reports published in English or Chinese. The search strategy combined the main keywords: neuroimaging, acupuncture, and depressive disorder. The strategy used to search PubMed is presented in **Table 1**; this strategy was modified to optimize searches through other databases. To find articles not detected in database searches, the authors (YO and LL) also searched the reference lists of relevant primary and review articles.

**Table 1:** Search strategy for the PubMed database.

#1	“Acupuncture Therapy”[MeSH Terms]
#2	(((((((((((((((((((Acupuncture, Ear[Title/Abstract]) OR (Meridians[Title/Abstract])) OR (Acupuncture Points[Title/Abstract])) OR (Moxibustion[Title/Abstract])) OR (Needling Methods[Title/Abstract])) OR (Ultrasound Acupuncture Therapy[Title/Abstract])) OR (Auricular Acupuncture[Title/Abstract])) OR (Body Acupuncture[Title/Abstract])) OR (Laser Acupuncture[Title/Abstract])) OR (Microneedle Acupuncture Therapy[Title/Abstract])) OR (Scalp Acupuncture[Title/Abstract])) OR (Abdominal Acupuncture Therapy[Title/Abstract])) OR (Fu's Acupuncture Therapy[Title/Abstract])) OR (Electric Stimulation Therapy[Title/Abstract])) OR (Transcranial Direct Current Stimulation[Title/Abstract])) OR (Transcutaneous Electric Nerve Stimulation[Title/Abstract])) OR (Vagus Nerve Stimulation[Title/Abstract])) OR (Electroacupuncture[Title/Abstract]))))
#3	#1 OR #2
#4	“Depressive Disorder”[MeSH Terms]
#5	(((((((((((((((((((((((((Depressive Disorders[Title/Abstract]) OR (Disorder, Depressive[Title/Abstract])) OR (Disorders, Depressive[Title/Abstract])) OR (Neurosis, Depressive[Title/Abstract])) OR (Depressive Neuroses[Title/Abstract])) OR (Depressive Neurosis[Title/Abstract])) OR (Neuroses, Depressive[Title/Abstract])) OR (Depression, Endogenous[Title/Abstract])) OR (Depressions, Endogenous[Title/Abstract])) OR (Endogenous Depression[Title/Abstract])) OR (Endogenous Depressions[Title/Abstract])) OR (Depressive Syndrome[Title/Abstract])) OR (Depressive Syndromes[Title/Abstract])) OR (Syndrome, Depressive[Title/Abstract])) OR (Syndromes, Depressive[Title/Abstract])) OR (Depression, Neurotic[Title/Abstract])) OR (Depressions, Neurotic[Title/Abstract])) OR (Neurotic Depression[Title/Abstract])) OR (Neurotic Depressions[Title/Abstract])) OR (Melancholia[Title/Abstract])) OR (Melancholias[Title/Abstract])) OR (Unipolar Depression[Title/Abstract])) OR (Depression, Unipolar[Title/Abstract])) OR (Depressions, Unipolar[Title/Abstract])) OR (Unipolar Depressions[Title/Abstract]))))
#6	#4 OR #5
#7	“Neuroimaging”[MeSH Terms]
#8	((((((((((((((((((((((((((((((((((Brain Imaging[Title/Abstract])) OR (Imaging, Brain[Title/Abstract])) OR (Diffusion Tensor Imaging[Title/Abstract])) OR (Imaging, Diffusion Tensor[Title/Abstract])) OR (Diffusion Tensor Magnetic Resonance Imaging[Title/Abstract])) OR (Diffusion Tensor MRI[Title/Abstract])) OR (Diffusion Tensor MRIs[Title/Abstract])) OR (MRI, Diffusion Tensor[Title/Abstract])) OR (DTI MRI[Title/Abstract])) OR (Diffusion Tractography[Title/Abstract])) OR (Tractography, Diffusion[Title/Abstract])) OR (Functional Neuroimaging[Title/Abstract])) OR (Neuroimaging, Functional[Title/Abstract])) OR (Functional Brain Imaging[Title/Abstract])) OR (Brain Imaging, Functional[Title/Abstract])) OR (Brain Imagings, Functional[Title/Abstract])) OR (Functional Brain Imagings[Title/Abstract])) OR (Imaging, Functional Brain[Title/Abstract])) OR (Imagings, Functional Brain[Title/Abstract])) OR (Brain Mapping[Title/Abstract])) OR (Mapping, Brain[Title/Abstract])) OR (Topographic Brain Mapping[Title/Abstract])) OR (Brain Mapping, Topographic[Title/Abstract])) OR (Mapping, Topographic Brain[Title/Abstract])) OR (Brain Electrical Activity Mapping[Title/Abstract])) OR (Functional Cerebral Localization[Title/Abstract])) OR (Functional Cerebral Localizations[Title/Abstract])) OR (functional magnetic resonance imaging[Title/Abstract])) OR (structural magnetic resonance imaging[Title/Abstract])) OR (fMRI[Title/Abstract])) OR (sMRI[Title/Abstract])) OR (Positron-Emission Tomography[Title/Abstract])) OR (PET[Title/Abstract])))
#9	#7 OR #8
#10	("2014/02/02"[Date - Create] : "3000"[Date - Create])
#11	#3 AND #6 AND #9 AND #10

### 2.3 Eligibility criteria

1. Participants: patients that were diagnosed with DD; the search was not restricted according to DD type.
2. Interventions: the acupuncture and moxibustion methods mainly include 1) manual acupuncture (MA); 2) percutaneous acupoint stimulation (transcutaneous electrical acupoint stimulation, TEAS), the TEAS mainly includes electroacupuncture (EA) and percutaneous acupoint electric nerve stimulation (transcutaneous electrical nerve stimulation, TENS) (33).
3. Comparisons: healthy controls (HC), placebo or sham acupuncture.
4. Study design: randomized and non-randomized controlled trials of using acupuncture to treat patients with DD.
5. At least one neuroimaging approach should have been used: structural magnetic resonance imaging (sMRI), functional magnetic resonance imaging (fMRI), functional near-infrared spectroscopy (fNIRS), positron emission tomography (PET), etc.

All neuroimaging clinical studies on the effects of using acupuncture to treat DD were eligible for inclusion. We excluded the experimental models, conference abstracts, case reports, protocols, animal studies, monographs, reviews, and studies lacking neuroimaging data.

### 2.4 Study selection and data extraction

The data extraction adhered to the Standards for Reporting Interventions in Clinical Trials of Acupuncture (STRICTA) guidelines. Details on the acupuncture interventions used in the reviewed studies were collected based on these guidelines. Two investigators (LL and PZ) independently extracted data from the included studies using a self-defined standardized extraction format. The extracted data included the name of the primary investigator, publication year, DD type, patient age, the country in which the study was performed, diagnosis, study design, type of acupuncture intervention, type of control intervention, the sample size of each intervention group, intervention duration, clinical variables, imaging modality, analysis methods, and brain imaging data. Any disagreements were solved by consensus between the two investigators. In the event that a consensus could not be reached, a third investigator (DP) made the final judgment. When information was missing from a study, the corresponding author was contacted if the information necessary for correspondence was available.

### 2.5 Risk-of-bias assessment

A Microsoft Excel template based on the Cochrane Handbook guidelines (available from https://www.riskofbias.info/) was used to independently assess the risk of bias in the extraction performed by two researchers (DL and YO). The risk of bias 2.0 tool (RoB 2) and the risk of bias in non-randomized studies of interventions tool (ROBINS-I) were used to evaluate the risk of bias of included randomized controlled trials (RCTs) and non-RCTs, respectively (34, 35). If disagreements were not resolved between the two reviewers, a third reviewer (SY) participated in the discussion until a consensus was reached.

### 2.6 Statistical analysis

Due to significant methodological differences among the included studies, we used descriptive statistical analysis to identify significant acupuncture-induced brain alterations in patients with DD as reported in the literature.

## 3 Results

### 3.1 Search results

**Figure 1:**
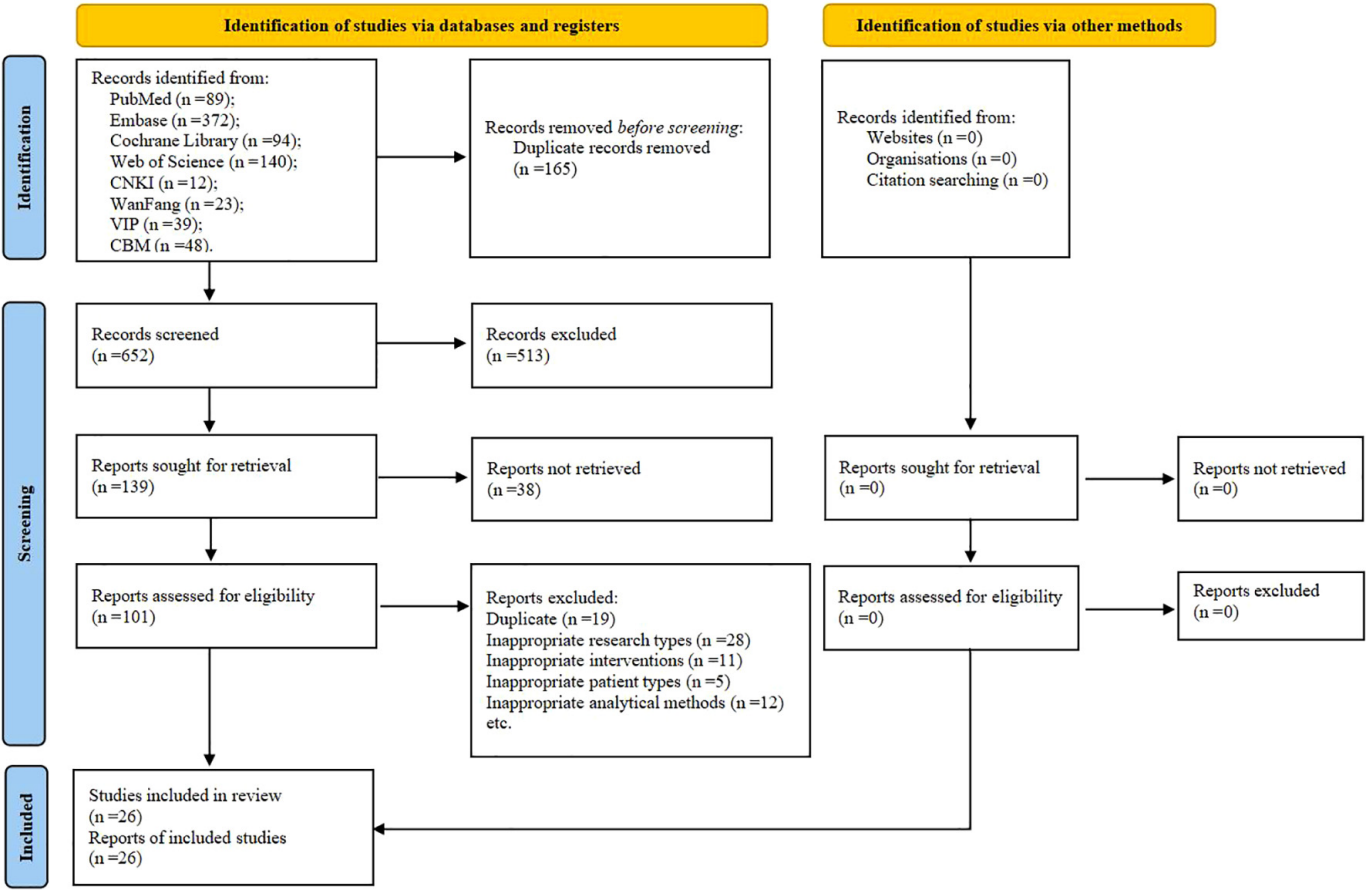
The PRISMA flow chart of selection process. CNKI, China National Knowledge Infrastructure; VIP, Chongqing VIP Database; CBM, Chinese Biomedical Literature Database.

A total of 817 potentially eligible articles were identified. After removing 165 duplicates and screening the 652 remaining articles, 139 candidate studies were isolated. Review of the full text of each shortlisted study based on the eligibility criteria excluded 113 more studies. Hence, 26 studies were included in this systematic review (25, 36–60) (**Figure 1**).

### 3.2 Study characteristics

The main characteristics of the included studies are shown in **Table 2**. The 26 studies (25, 36–60) included 1138 participants, with a total of 742 DD patients and 396 HCs. All studies were non-RCTs and came from China.

**Table 2:** Detailed characteristics of included trials.

Study	Study design	Severity	Number	Age (Years) (I/C1/C2)	Gender (M/F)		Diagnostic criteria	Intervention group	Control group	Treatment duration
					I	C				
He JK et al., 2023 (36)	Non-RCT	Major	45	43.05±14.90/ 44.04±0.44	4/18	5/18	DSM-V	taVNS	HC	8w
Sun JF et al., 2023a (37)	Non-RCT	Major	109	30.69 ± 8.33/31.64±8.40	17/38	17/37	DSM-V	taVNS	HC	Immediate
Sun JF et al., 2023b (38)	Non-RCT	FED	58	35±14/36±13	5/24	7/22	DSM-V	taVNS	HC	Immediate
Cao DN et al., 2019 (39)	Non-RCT	Late-life depression	15	67.0 ±3.5/—	15/0	—	DSM-IV	MA	—	Immediate
Ma Y et al., 2023 (40)	Non-RCT	—	64	40±11/38±13	13/19	11/21	DSM-V	taVNS	HC	8w
Ma Y et al., 2022 (41)	Non-RCT	TRD	80	43.01±11.90/38.33±13.04	16/24	13/27	DSM-IV or DSM-V	taVNS	HC	Immediate
Sun JF et al., 2022a (42)	Non-RCT	TRD	90	44.13±12.02/41.89±12.91	19/25	18/28	DSM-V	taVNS	HC	Immediate
Sun JF et al., 2022b (43)	Non-RCT	TRD	60	40.7±11.8/40.5±13.0	8/22	8/22	Refer to the TRD diagnostic criteria proposed by Gaynes BN	taVNS	HC	Immediate
Sun JF et al., 2022c (44)	Non-RCT	Recurrent	60	40.7±11.8/40.5±13.0	8/22	8/22	DSM-V	taVNS	HC	Immediate
Yi SJ et al., 2022 (45)	Non-RCT	Major	20	27.70±8.48/—	5/15	—	DSM-IV	taVNS	—	4w
Wang Z et al., 2022 (46)	Non-RCT	TRD	68	44.47±11.80/29.50±23.00	16/18	7/27	Refer to the TRD diagnostic criteria proposed by Sackeim	taVNS	HC	Immediate
Chen LM et al., 2022 (47)	Non-RCT	Recurrent	22	NR	NR	—	DSM-IV	taVNS	—	8w
Chen LM et al., 2021 (48)	Non-RCT	TRD	24	NR	NR	—	Refer to the TRD diagnostic criteria proposed by Sackeim	taVNS	—	8w
Wei XY et al., 2021 (49)	Non-RCT	Mild	20	26.45±1.82/—	10/10	—	DSM-IV	EA	—	Immediate
Duan GX et al., 2020 (50)	Non-RCT	Major	58	28.69±6.69/26.76±1.72	9/20	15/14	DSM-V	EA	HC	Immediate
Li XJ et al., 2020 (51)	Non-RCT	TRD	17	45.59±9.15/—	9/8	—	DSM-V and Refer to the TRD diagnostic criteria proposed by Sackeim	taVNS	—	8w
Chen HY et al., 2019 (52)	Non-RCT	Mild and moderate	20	41.95±15.26/—	3/17	—	ICD-10	taVNS	—	8w
Tu YH et al., 2018 (53)	Non-RCT	Mild and moderate	41	NR	NR	—	ICD-10	tVNS	stVNS	4w
Wang ZJ et al., 2018 (54)	Non-RCT	Major	37	40.85±11.05/40.35±13.29	5/12	7/13	ICD-10	tVNS	stVNS	4w
Fang JL et al., 2016a (55)	Non-RCT	Major	38	40.35/40.38	5/12	7/14	ICD-10	tVNS	stVNS	4w
Fang JL et al., 2016b (56)	Non-RCT	Major	34	39.17±14.85/42.56±10.64	5/13	5/11	ICD-10	tVNS	stVNS	4w
Liu J et al., 2016 (57)	Non-RCT	Major	34	37.83±6.46/42.56±10.64	6/12	5/11	ICD-10	tVNS	stVNS	5w
Han HY et al., 2016 (58)	Non-RCT	Mild	14	NR	NR	—	CCMD-3	MA	—	Immediate
Deng DM et al., 2016 (25)	Non-RCT	Major	58	28.69±6.69/26.76±1.72	9/20	15/14	DSM-IV	EA	HC	Immediate
Deng DM et al., 2015a (59)	Non-RCT	Major	36	26.88±4.10/25.30±1.38	6/10	10/10	DSM-IV	EA	HC	Immediate
Deng DM et al., 2015b (60)	Non-RCT	Major	16	NR	6/10	NR	DSM-IV	EA	sEA	Immediate

FED, first-episode depression; TRD, treatment-resistant depression; I, intervention group; C, control group; M, male; F, female; NR, not reported; ICD, International Classification of Disease; CCMD, Chinese Classification of Mental Disorders; DSM, Diagnostic and Statistical Manual; MA, manual acupuncture; taVNS, transcutaneous auricular vagus nerve stimulation; TECAS, transcutaneous electrical cranial-auricular acupoints stimulation; EA, electroacupuncture; tVNS, transcutaneous vagus nerve stimulation; HC, healthy control; sEA, sham electroacupuncture; stVNS, sham transcutaneous vagus nerve stimulation; d, day; w, week.

Regarding the diagnostic criteria for DD, 16 studies used the DSM-IV or DSM-V criteria (25, 36–42, 44, 45, 47, 49–51, 60), four studies referred to the TRD diagnostic criteria proposed by Sackeim (43, 46, 48, 51), ICD-10 was used in six studies (52–57), and CCMD-3 was used in one study (58).

In terms of DD types, MDD was diagnosed in 11 studies (25, 36, 37, 45, 50, 54–57, 59, 60), first-episode depression was diagnosed in one study (38), late-life depression was diagnosed in one study (39), treatment-resistant depression was diagnosed in six studies (41–43, 46, 48, 51), recurrent depression was diagnosed in two studies (44, 47), mild and/or moderate depression was diagnosed in four studies (49, 52, 53, 58), and the remaining were not specified.

All included studies used at least one of the following acupuncture techniques: transcutaneous auricular vagus nerve stimulation (taVNS) (36–38, 40–48, 51, 52), transcutaneous vagus nerve stimulation (tVNS) (53–57), EA (25, 49, 50, 59, 60), MA (39, 58). 12 studies (25, 36–38, 40–44, 46, 50, 59) used HCs to populate a control group, six (53–57, 60) used sham-acupuncture group as their controls, and the remaining were not specified.

In terms of the selection of stimulation sites, the auricular points for the heart and kidney (38, 40, 43, 44, 46–48, 51, 52), the auricular concha area (36, 37, 41, 42, 45, 53–57), and Baihui (GV20) (25, 49, 50, 59, 60) were selected by most studies. Most studies observed acupuncture retention times of 20–30 min.

### 3.3 Clinical variables

To evaluate depressive disorder, 17 studies used the Hamilton Rating Scale for Depression (HAMD) series scale. The 17-item HAMD was used in 12 (36–38, 40, 41, 43, 45–48, 51, 52), the 24-item HAMD in four (53, 55–57), and an unspecified version of the HAMD in one (54). Four studies used the 17-item hamilton depression rating scale (HDRS-17) (25, 45, 49, 50). 11 studies evaluated their patients using the Hamilton Anxiety Rating Scale (HAMA). The 14-item HAMA was used in five (36, 40, 43, 46, 53), and the HAMA in six (38, 41, 47, 55–57). Nine studies used the Self-Rating Depression Scale (SDS) and the Self-Rating Anxiety Scale (SAS) (25, 45–47, 50, 53, 55–57). One study used the ruminative response scale (RRS) (47). Two studies used the wisconsin card sorting test (WCST) (36, 40). One study used Visual Analysis Scale (VAS) (49).

### 3.4 Imaging modality and analysis

All 26 studies used magnetic resonance imaging (MRI) to explore neural changes following the administration of acupuncture (**Table 3**). Of the 26 studies that evaluated changes in neuronal activity, 24 used resting state functional magnetic resonance imaging (rs-fMRI) (25, 36–52, 54, 56–60) and two studies applied functional magnetic resonance imaging (fMRI) (53, 55) to evaluate the amplitude of low frequency fluctuation (ALFF), functional connectivity (FC), regional homogeneity (ReHo), resting state functional connectivity (rs-FC), independent component analysis (ICA), degree centrality (DC), and brain activation.

**Table 3:** The neuroimage information of the included studies.

Study	Clinical variables	Examination time	Scan T	Imaging modality	Analytical Methods	Neuroimaging results
**He JK et al., 2023 (** 36)	HAMD-17, HAMA-14, WCST	Before and after 8 weeks of treatment	3.0T	rs-fMRI	FC	**After taVNS treatment:** ↓FC: The inferior ventral striatum and precuneus; **Correlation analysis:** Positive correlations were found between the HAM-D remission rate and changes in brain activity in the inferior ventral striatum.
**Sun JF et al., 2023a (** 37)	HAMD-17	Before and after treatment	3.0T	rs-fMRI	ALFF, FC	**After taVNS treatment:** ↓ALFF: Right precuneus; ↓FC: Right precuneus, left middle frontal gyrus, the left posterior cingulate gyrus and the left angular gyrus; **Correlation analysis:** The FC values between the right precuneus and left posterior cingulate gyrus in the pre-treatment MDD group were negatively correlated with HAMD-17 scores.
**Sun JF et al., 2023b (** 38 **)**	HAMD-17, HAMA	Before and after treatment	3.0T	rs-fMRI	ALFF, FC	**vs HC**: ↑ALFF: Left middle frontal gyrus, right middle frontal gyrus, the left orbital area of the Inferior frontal gyrus, and right middle temporal gyrus; ↓ALFF: Left precuneus, right precuneus, left rectangular gyrus, right rectangular gyrus, and left putamen. **After taVNS treatment:** ↓ALFF: Left middle frontal gyrus, left supplementary motor area; ↑FC: Right precuneus, left rectangular gyrus, right superior occipital gyrus, and right central sulcus tegmental gyrus; ↓ FC: Left middle frontal gyrus and the left orbital area of the Inferior frontal gyrus.
**Cao DN et al., 2019 (** 39)	—	Before and after treatment	3.0T	rs-fMRI	ReHo	**After acquisition treatment:** ↓ReHo: Left middle temporal gyrus, left inferior temporal gyrus, right inferior occipital gyrus, right middle temporal gyrus, the left orbital area of the Inferior frontal gyrus, left occipital gyrus, left angular gyrus, bilateral superior frontal gyrus, bilateral middle frontal gyrus, left cuneiform lobe, and superior parietal lobule; ↑ReHo: Left inferior temporal gyrus, left insula, bilateral hippocampus, and parahippocampal gyrus.
**Ma Y et al., 2023 (** 40)	HAMD-17, HAMA-14, WCST	Before and after 8 weeks of treatment	3.0T	rs-fMRI	DC	**vs HC**: ↑DC: occipital lobe, parietal lobe, and limbic system; ↓DC: Sensory motor cortex and temporal lobe; **After taVNS treatment:** ↓DC: Left insula, left auxiliary motor area, right putamen; ↑DC: Left cerebellar peduncle region 1, right auxiliary motor area, right middle frontal gyrus, and left inferior temporal gyrus. **Correlation analysis:** After treatment, the DC value of the left auxiliary motor area in patients with depression was negatively correlated with HAMD and HAMA scores. The DC value of the left cerebellar peduncle region 1 is negatively correlated with the WCST total response score. The DC value of the left insula is positively correlated with the WCST total response score.
**Ma Y et al., 2022 (** 41)	HAMD-17, HAMA	30 minutes before and after treatment	3.0T	rs-fMRI	ReHo, FC	**After taVNS treatment:** ↓ReHo: Medial orbitofrontal cortex; **Correlation analysis:** ANCOVA of the mOFC-Based FC images revealed a significant interaction effect on the left inferior parietal gyrus and left superior marginal gyrus.
**Sun JF et al., 2022a (** 42)	—	Before and after treatment	3.0T	rs-fMRI	ALFF, FC	**vs HC:** ↑ALFF: The left orbital area of the middle frontal gyrus. **After taVNS treatment:** ↓ALFF: The left orbital area of the middle frontal gyrus and right middle frontal gyrus; ↑ALFF: The right orbital area of the superior frontal gyrus; ↑FC: The left orbital area of the middle frontal gyrus with left middle frontal gyrus and the right inferior occipital gyrus.
**Sun JF et al., 2022b (** 43)	HAMD-17, HAMA-14	Before and after treatment	3.0T	rs-fMRI	ReHo, FC	**vs HC**: ↑ReHo: Left putamen/left amygdala, left straight gyrus/left anterior cingulate gyrus; ↓ReHo: Right caudate nucleus, left talus sulcus, left precuneus/left paracentral lobule, right middle temporal gyrus and left cerebellarvermis region 2; **vs HC, the right caudate nucleus is the region of interest:** ↑FC: Left precuneus/right precuneus, left middle occipital gyrus; **After taVNS treatment, the right caudate nucleus is the region of interest:** ↓FC: Left precuneus/right precuneus, left superior occipital gyrus.
**Sun JF et al., 2022c (** 44)	—	Before and after treatment	3.0T	rs-fMRI	ALFF	**vs HC:** ↑ALFF: Left medial superior frontal gyrus/left medial orbital superior frontal gyrus, right angular gyrus/right middle occipital gyrus; ↓ALFF: Bilateral precentral gyrus, left precuneus/left paracentral lobule; **After taVNS treatment:** ↑ALFF: Right precentral gyrus, left precuneus/left paracentral lobule; ↓ALFF: Left anterior cingulate gyrus/left medial orbital superior frontal gyrus, left angular gyrus/left middle occipital gyrus.
**Yi SJ et al., 2022 (** 45)	HDRS-17, HAMA-17, SDS and SAS	Before and after 4 weeks of treatment	3.0T	rs-fMRI	ReHo	**After taVNS treatment:** ↓ReHo: The left/right median cingulate cortex, the left precentral gyrus, the left postcentral gyrus, the right calcarine cortex, the left supplementary motor area, the left paracentral lobule, and the right lingual gyrus. **Correlation analysis:** Pearson analysis revealed a positive correlation between changes of ReHo in the right median cingulate cortex/the left supplementary motor area and changes of HAMD scores after taVNS.
**Wang Z et al., 2022 (** 46)	HAMD-17, HAMA-14, SDS and SAS	Before and after treatment	3.0T	rs-fMRI	ALFF, FC	**vs HC**: ↑ALFF: Left globus pallidus/hypothalamus/caudate nucleus, left precentral gyrus/middle frontal gyrus; ↑FC: Left precentral gyrus/middle frontal gyrus, bilateral cingulate gyrus, bilateral auxiliary motor areas, left medial superior frontal gyrus, left lenticular nucleus (including globus pallidus and putamen); **After taVNS treatment:** ↑ALFF: Left orbital inferior and middle frontal gyrus, bilateral cerebellar region 1, and left cerebellar region 2; **Correlation analysis:** The left precentral gyrus/middle frontal gyrus ALFF values were positively correlated with HAMD-17 and SDS scores, respectively.
**Chen LM et al., 2022 (** 47)	HAMD-17, HAMA, SDS, SAS, and RRS	Before and after 8 weeks of treatment	3.0T	rs-fMRI	FC	**After taVNS treatment:** ↓FC: The left globus pallidus and the right postcentral gyrus, inferior parietal gyrus, the right globus pallidus and the left superior marginal gyrus, postcentral gyrus, superior parietal gyrus, inferior parietal gyrus, precuneus, right postcentral gyrus, superior marginal gyrus, and inferior parietal gyrus, between the right caudate and the right lingual gyrus, calcarine gyrus, and cerebellum. **Correlation analysis:** Changes in FC between the right globus pallidus and the left inferior parietal gyrus, between the left globus pallidus and the right postcentral gyrus were negatively correlated with HAMD-17 scores change before and after treatment. The change of FC between the right globus pallidus and the right postcentral gyrus was negatively correlated with the change in SDS. The difference in FC between the right globus pallidus and the right postcentral gyrus was negatively correlated with the change in SAS.
**Chen LM et al., 2021 (** 48)	HAMD-17	Before and after 8 weeks of treatment	3.0T	rs-fMRI	ALFF, ReHo, rs-FC	**After taVNS treatment:** ↓ALFF: Right insula and putamen; ↓ReHo: Right insula, putamen, anterior cingulate gyrus, caudate nucleus, thalamus; ↓rs-FC: Right insula, left superior frontal gyrus and middle frontal gyrus; **Correlation analysis:** There is a significant negative correlation between the ReHo difference in the right insula and the reduction rate of HAMD-17. The rs-FC difference between the right insula and the left superior frontal gyrus was significantly negatively correlated with the HAMD-17 score reduction rate.
**Wei XY et al., 2021 (** 49)	HDRS-17, VAS	Phase I: before needle insertion; Phase II: during EA; Phase III: 15 minutes after needle removal.	3.0T	rs-fMRI	ReHo	**During EA treatment vs Before EA treatment:** ↑ReHo: Bilateral postcentral gyrus, right calcarine gyrus, and right cuneus; **After EA treatment vs During EA treatment:** ↓ReHo: Right precuneus, posterior cingulate cortex, and right angular gyrus; **After EA treatment vs Before EA treatment:** ↑ReHo: Right calcarine gyrus, right cuneus, and bilateral postcentral gyrus; ↓ReHo: Right posterior cingulate cortex, right angular gyrus, and right precuneus; **Correlation analysis:** The ReHo values of right postcentral gyrus and left postcentral gyrus in after EA treatment was positively correlated with the VAS scores. The ReHo values of right posterior cingulate cortex, right angular gyrus, right precuneus in after EA treatment was negatively correlated with VAS scores. The ReHo values of right postcentral gyrus and left postcentral gyrus in after EA treatment was positively correlated with the HDRS scores. The ReHo values of right angular gyrus in before EA treatment was positively correlated with the HDRS scores.
**Duan GX et al., 2020 (** 50)	HDRS-17, SDS, SAS	Before and after treatment	3.0T	rs-fMRI	FC	**vs HC:** ↑FC: Between amygdala and hippocampus, precuneus, precentral gyrus and angular gyrus; ↓FC: Between amygdala and orbitofrontal cortex; **After EA treatment:** ↓FC: Bilateral precentral gyrus, periaqueductal gray, left anterior insula, right posterior insula, right precuneus and right dorsal anterior cingulate cortex with left amygdala; ↑FC: Right orbitofrontal cortex and left dorsolateral prefrontal cortex with the left amygdala; ↓FC: Between the right amygdala and periaqueductal gray, bilateral precentral gyrus, right posterior insula; ↑FC: Between right amygdala and bilateral orbitofrontal cortex and left dorsolateral prefrontal cortex.
**Li XJ et al., 2020 (** 51)	HAMD-17	Before and after 8 weeks of treatment	3.0T	rs-fMRI	rs-FC	**After taVNS treatment, the rACC as seed point:** ↑FC: Right rACC-left precuneus/left postcentral gyrus, left rACC-left lingual gyrus/left precuneus/left postcentral gyrus; ↓FC: Left rACC-right precuneus; **Correlation analysis:** There is a significant positive correlation between the enhanced FC of left rACC-left lingual gyrus and the difference in HAMD-17 scores.
**Chen HY et al., 2019 (** 52)	HAMD-17	Before and after 8 weeks of treatment	3.0T	rs-fMRI	ALFF	**After taVNS treatment:** ↑ALFF: Left inferior temporal gyrus; ↓ALFF: Right hippocampus, precuneus, cortex around the calcarine fissure, left posterior cingulate gyrus, and right thalamus.
**Tu YH et al., 2018 (** 53)	HAMD-24, HAMA-14, SDS and SAS	Before and after 4 weeks of treatment	1.5T	fMRI	FC	**tVNS group vs stVNS group:** ↓FC: Between the bilateral medial hypothalamus (MH) and rostral anterior cingulate cortex (rACC) was significantly decreased during tVNS but not during sham tVNS. **Correlation analysis:** The strength of this FC was significantly correlated with HAM-D improvements after 4 weeks of tVNS.
**Wang ZJ et al., 2018 (** 54)	HAMD	Before and after 4 weeks of treatment	1.5T	rs-fMRI	FC	**tVNS group vs stVNS group:** ↑FC: Between the left NAc and bilateral MPFC/rACC in the slow-5 band (0.008-0.027) and between the right NAc and left insula, occipital gyrus, and right lingual/fusiform gyrum in the typical low band (0.008-0.09); **Correlation analysis:** The FC of the NAc-MPFC/rACC during real tVNS showed a negative association with HAMD score changes in the real tVNS group after one month of treatment.
**Fang JL et al., 2016a (** 55)	HAMD-24, HAMA, SDS and SAS	Before and after 4 weeks of treatment	1.5T	fMRI	brain activation	**tVNS group:** Increased activation: Left insula and bilateral cerebellum; Deactivation: DMN(MPFC and precuneus); **stVNS group:** Increased activation: Right inferior frontal gyrus; Deactivation: DMN(MPFC, posterior cingulate gyrus, precuneus), left hippocampus; **Correlation analysis:** A significant negative correlation between HAM-D scores at 4 weeks and activation in the left dorsal anterior insula during the first treatment session in the tVNS group.
**Fang JL et al., 2016b (** 56)	HAMD-24, HAMA, SDS, SAS	Before and after 4 weeks of treatment	1.5T	rs-fMRI	ICA, FC	**After tVNS treatment:** ↓FC: Between the DMN and anterior insula and parahippocampus; **vs stVNS:** ↑FC: Between the DMN and precuneus and orbital prefrontal cortex; **Correlation analysis:** All these FC increases are also associated with 24-item Hamilton Depression Rating Scale reduction.
**Liu J et al., 2016 (** 57)	HAMD-24, HAMA, SDS, SAS	Before and after 4 weeks of treatment	1.5T	rs-fMRI	rs-FC	**vs stVNS, after tVNS treatment:** ↑rsFC: Right amygdala and left dorsolateral prefrontal cortex; **Correlation analysis:** All the rsFC increases were also associated with HAMD reduction as well as reductions in the anxiety and retardation HAMD subscales.
**Han HY et al., 2016 (** 58)	—	Before and after treatment	—	rs-fMRI	FC	**After MA treatment, take amygdala as seed point:** ↓FC: Right superior frontal gyrus, middle frontal gyrus.
**Deng DM et al., 2016(** 25)	SDS, SAS, HDRS-17	Before and after treatment	3.0T	rs-fMRI	FC	**After EA treatment:** ↑FC: Precuneus/posterior cingulate cortex and bilateral anterior cingulate cortex; ↓FC: Precuneus/posterior cingulate cortex and left middle prefrontal cortex, left angualr gyrus and bilateral hippocampus/parahippocampus.
**Deng DM et al., 2015a (** 59)	—	Before and after treatment	3.0T	rs-fMRI	ALFF	**vs HC:** ↑ALFF: Right anterior cingulate gyrus, middle cingulate gyrus, lentiform nucleus, precuneus, and bilateral insula; ↓ALFF: Bilateral hippocampus, cerebellum, and occipital lobe; **After electroacupuncture treatment:** ↓ALFF: Right anterior and middle cingulate gyrus, lenticular nucleus, and left insula; ↑ALFF: Left hippocampus and cerebellum.
**Deng DM et al., 2015b (** 60)	—	Before and after treatment	3.0T	rs-fMRI	ReHo	**After EA treatment vs After sEA treatment:** ↑ReHo: Left ventromedial prefrontal lobe, left insular lobe, left anterior cingulate gyrus, right thalamus, right superior temporal gyrus, bilateral precuneus, bilateral anterior lobe of cerebellum and right posterior cerebellar lobe.

HAMD, hamilton rating scale for depression; WCST, wisconsin card sorting test; RRS, ruminative response scale; HAMA, hamilton anxiety rating scale; SDS, self rating depression scale; SAS, self rating anxiety scale; VAS, visual analogue scale; HDRS, hamilton depression rating scale; rs-fMRI, resting state functional magnetic resonance imaging; fMRI, functional magnetic resonance imaging; ALFF, amplitude of low frequency fluctuation; ReHo, regional homogeniety; rs-FC, resting state functional connectivity; FC, functional connectivity; DC, degree centrality; ICA, independent component analysis; GCA, granger causality analysis; taVNS, transcutaneous auricular vagus nerve stimulation; HC, healthy control; rACC, rostral anterior cingulate cortex; EA, electroacupuncture; sEA, sham electroacupuncture; MA, manual acupuncture; DD, depressive disorder; tVNS, transcutaneous vagus nerve stimulation; stVNS, sham transcutaneous vagus nerve stimulation; DMN, default mode network; mOFC, medial orbitofrontal cortex; ANOVA, analysis of variance; MDD, major depressive disorder; NAc, nucleus accumbens; MPFC, medial prefrontal cortex.

### 3.5 Results of risk of bias assessment

Of the 26 non-RCTs (25, 36–60), 11 had a “Low” overall risks of bias (38–41, 43, 44, 46, 52, 53, 59, 60). The remaining 15 had “Serious” risks of bias due to participant dropout (25, 36, 37, 42, 45, 47–51, 54–58). A summary of the risk of bias assessment of each included study is presented in **Table 4**.

**Table 4:** The results of risk of bias assessment of non-randomised controlled trials by the ROBINS-I.

Study	Bias due to confounding	Bias in selection of participants into study	Bias in classification of interventions	Bias due to deviations from intended interventions	Bias due to missing data	Bias in measurement of outcomes	Bias in selection of the reported result	Overall risk of bias
He JK et al., 2023 (31)	Low	Low	Low	Low	Serious	Low	Low	Serious
Sun JF et al., 2023a (32)	Low	Low	Low	Low	Serious	Low	Low	Serious
Sun JF et al., 2023b (33)	Low	Low	Low	Low	Low	Low	Low	Low
Cao DN et al., 2019 (34)	Low	Low	Low	Low	Low	Low	Low	Low
Ma Y et al., 2023 (35)	Low	Low	Low	Low	Low	Low	Low	Low
Ma Y et al., 2022 (36)	Low	Low	Low	Low	Low	Low	Low	Low
Sun JF et al., 2022a (37)	Low	Low	Low	Low	Serious	Low	Low	Serious
Sun JF et al., 2022b (38)	Low	Low	Low	Low	Low	Low	Low	Low
Sun JF et al., 2022c (39)	Low	Low	Low	Low	Low	Low	Low	Low
Yi SJ et al., 2022 (40)	Low	Low	Low	Low	Serious	Low	Low	Serious
Wang Z et al., 2022 (41)	Low	Low	Low	Low	Low	Low	Low	Low
Chen LM et al., 2022 (42)	Low	Low	Low	Low	Serious	Low	Low	Serious
Chen LM et al., 2021 (43)	Low	Low	Low	Low	Serious	Low	Low	Serious
Wei XY et al., 2021 (44)	Low	Low	Low	Low	Serious	Low	Low	Serious
Duan GX et al., 2020 (45)	Low	Low	Low	Low	Serious	Low	Low	Serious
Li XJ et al., 2020 (46)	Low	Low	Low	Low	Serious	Low	Low	Serious
Chen HY et al., 2019 (47)	Low	Low	Low	Low	Low	Low	Low	Low
Tu YH et al., 2018 (48)	Low	Low	Low	Low	Low	Low	Low	Low
Wang ZJ et al., 2018 (49)	Low	Low	Low	Low	Serious	Low	Low	Serious
Fang JL et al., 2016a (50)	Low	Low	Low	Low	Serious	Low	Low	Serious
Fang JL et al., 2016b (51)	Low	Low	Low	Low	Serious	Low	Low	Serious
Liu J et al., 2016 (52)	Low	Low	Low	Low	Serious	Low	Low	Serious
Han HY et al., 2016 (53)	Low	Low	Low	Low	Serious	Low	Low	Serious
Deng DM et al., 2016 (54)	Low	Low	Low	Low	Serious	Low	Low	Serious
Deng DM et al., 2015a (55)	Low	Low	Low	Low	Low	Low	Low	Low
Deng DM et al., 2015b (56)	Low	Low	Low	Low	Low	Low	Low	Low

### 3.6 Brain imaging data

#### 3.6.1 Brain alterations of DD

Compared with HCs, patients with DD exhibited aberrant activity in the precuneus (25, 36–38, 43, 44, 50, 59), cingulate gyrus (37, 43, 44, 46, 50, 59), middle frontal gyrus (37, 38, 40, 42, 46), cerebellum (40, 43, 46, 59), and angular gyrus (25, 37, 44, 50).

A correlation analysis was conducted of pre-treatment neural activity associated with DD (37). The FC values between the right precuneus and left posterior cingulate gyrus in the pre-treatment MDD group were negatively correlated with HAMD-17 scores.

#### 3.6.2 Acupuncture-related brain alterations in patients with DD

In the 14 studies using transcutaneous auricular vagus nerve stimulation (taVNS) (36–38, 40–48, 51, 52), the most common neural changes in patients with DD following the intervention were observed in the precuneus (36–38, 43, 44, 47, 51, 52), cingulate gyrus (37, 43, 44, 46, 48, 51, 52), middle frontal gyrus (37, 38, 40, 42, 46, 48), putamen (38, 40, 43, 46, 48), and cerebellum (40, 43, 46, 47).

Of the five studies that used transcutaneous vagus nerve stimulation (tVNS) (53–57), the most frequent neural changes in patients DD patients following the intervention were observed in the prefrontal lobe (54–57), insula (54–56), cingulate gyrus (54, 55), precuneus (55, 56).

In the five studies that employed EA (25, 49, 50, 59, 60), the most common neural changes in patients with DD following the intervention were observed in the precuneus (25, 49, 50, 59, 60), cingulate gyrus (50, 59, 60), insula (50, 59, 60), prefrontal lobe (25, 50, 60), angular gyrus (25, 49, 50), hippocampus (25, 50, 59).

Two studies found that the superior frontal and middle frontal gyri of patients with DD exhibited changes following the administration of MA (39, 58).

#### 3.6.3 Correlation between the neuroimaging results and clinical variables

The neuroimaging findings in most patients with DD were correlated with their HAMD scores. One study found that positive correlations were found between the HAM-D remission rate and changes in brain activity in the inferior ventral striatum (36). Another investigation found that the FC values between the right precuneus and left posterior cingulate gyrus in the pre-treatment MDD group were negatively correlated with HAMD-17 scores (37). Another study found that after treatment, the DC value of the left auxiliary motor area in patients with depression was negatively correlated with HAMD scores (40). A study (45) reported Pearson analysis revealed a positive correlation between changes of ReHo in the right median cingulate cortex/the left supplementary motor area and changes of HAMD scores after taVNS. One study found that the left precentral gyrus/middle frontal gyrus ALFF values were positively correlated with HAMD-17 scores, respectively (46). Another trail found that changes in FC between the right globus pallidus and the left inferior parietal gyrus, between the left globus pallidus and the right postcentral gyrus were negatively correlated with HAMD-17 scores change before and after treatment (47). Another study reported there is a significant negative correlation between the ReHo difference in the right insula and the reduction rate of HAMD-17, the rs-FC difference between the right insula and the left superior frontal gyrus was significantly negatively correlated with the HAMD-17 score reduction rate (48). Another study found that there is a significant positive correlation between the enhanced FC of left rACC-left lingual gyrus and the difference in HAMD-17 scores (51). Another study reported the strength of this FC was significantly correlated with HAM-D improvements after 4 weeks of tVNS (53). Another study found that the FC of the NAc-MPFC/rACC during real tVNS showed a negative association with HAMD score changes in the real tVNS group after one month of treatment (54). A study reported a significant negative correlation between HAM-D scores at 4 weeks and activation in the left dorsal anterior insula during the first treatment session in the tVNS group (55). Another study found that all the rsFC increases were also associated with HAMD reduction as well as reductions in the anxiety and retardation HAMD subscales (57).

In addition, the neuroimaging results of patients with DD were found by several studies to be related to HDRS, HAMA, SDS, SAS, VAS, and the WCST total response scores (**Table 3**).

#### 3.6.4 Neural regions most affected by acupuncture in patients with DD

Acupuncture-induced brain changes in patients with DD were mainly observed in the cingulate gyrus (25, 37, 40, 43–46, 48–55, 59, 60), precuneus (25, 36–38, 43, 44, 47, 49–52, 55, 56, 59, 60), insula (39, 40, 48, 50, 54–56, 59, 60), prefrontal lobe (25, 41, 50, 54–57, 60), middle frontal gyrus (37–40, 42, 46, 48, 58), cerebellum (40, 43, 46, 47, 55, 59, 60), hippocampus (25, 39, 40, 50, 52, 55, 59), putamen (38, 40, 43, 46, 48, 59), angular gyrus (25, 37, 39, 44, 49, 50), superior frontal gyrus (39, 44, 46, 48, 58), lingual gyrus (45, 47, 51, 54), precentral gyrus (44–46, 50), postcentral gyrus (45, 47, 49, 51), thalamus (40, 48, 52, 60), parahippocampal gyrus (25, 39, 40, 56), and amygdala (40, 43, 50, 57).

## 4 Discussion

### 4.1 Study characteristics of the included studies

As far as we know, as the latest and most comprehensive systematic review, this review summarizes the neuroimaging research on acupuncture treatment of DD in the past decade. It included 742 patients with DD and 396 HCs.

In terms of study design, this review evaluated a total of 26 non-RCTs from China, with sample sizes ranging from 14 to 109 individuals per study. None of the studies mentioned randomization and allocation concealment, and only three studies involved blinding (blinding participants) (54, 56, 57). According to the bias risk assessment results of non-RCTs, 15 studies had “Serious” risks of bias due to participant dropout (25, 36, 37, 42, 45, 47–51, 54–58). In addition, RCTs are the gold standard for comparing intervention effects and effectiveness in medical research design (61). Therefore, in order to reduce the risk of bias and enhance the credibility of the study, more careful design is needed in future research on random methods and allocation concealment, blinding, sample size evaluation, and processing of subject dropout data.

In terms of intervention details, the involved acupuncture methods include taVNS, tVNS, EA and MA, which can change the function of brain regions related to DD patients, thus alleviating their mental and physical symptoms. In addition, taVNS, tVNS and EA seem to be the most commonly used acupuncture techniques in the included studies. The auricular points of the heart and kidney, auricular concha, and Baihui (GV20) are the most frequently used stimulation sites. This may be because the surface of the ear is distributed with the afferent vagus nerve, and the surface distribution of the auricular concha or auricular points and the vagus nerve ear branch mostly overlap (62). The anatomical pathway of the vagus nerve is connected to many brain regions related to emotion regulation, such as the amygdala, hippocampus, cingulate gyrus, prefrontal lobe, and insula (63). Therefore, acupuncture stimulation of the auricle or auricular points can activate the vagus nerve pathway, regulate the above brain regions, and alleviate depressive symptoms. Furthermore, vagus nerve stimulation is associated with the neural plasticity of brain networks. Vagus nerve stimulation can enhance the neural plasticity of related brain regions such as the hippocampus to regulate the emotional and cognitive functions of DD (64). In addition, the onset of DD has been associated with reduced levels of monoamine neurotransmitters such as 5-hydroxytryptamine (5-HT) and dopamine (DA) in the central nervous system (65), as well as cytokines such as interleukin-6 (IL-6) and IL-1β (66). Anti-inflammatory cytokines, including IL-10 and IL-4, may exert antidepressant effects (67). Acupuncture stimulation of GV20 can reduce serum concentrations of IL-6 and IL-1β in patients with DD, while increasing the serum levels of IL-10 and IL-4 (68, 69), as well as the expression of 5-HT and DA in the hippocampus and hypothalamus (70). Overall, molecular changes help to attenuate the depressive state. However, the included research lacks the comparison of different acupuncture methods and stimulation sites, so it is impossible to recommend the preferred acupuncture techniques and stimulation sites for clinical practice. We hope that future research can pay attention to this.

In terms of control setting, 12 studies (25, 36–38, 40–44, 46, 50, 59) compared patients with HC and DD, aiming to explore the neuropathological characteristics of DD. It was found that patients with DD exhibited neuroimaging abnormalities in certain brain regions, such as the precuneus, cingulate gyrus, middle frontal gyrus, cerebellum, and angular gyrus. Six trails (53–57, 60) used the sham acupuncture group as a control to exclude any changes caused by perception of receiving real acupuncture. This study found that real acupuncture could activate more relevant brain regions in patients with DD than sham acupuncture, and could increase the correlation with clinical variables.

In terms of the outcome measures, the included studies mainly used the HAMD series of scales to evaluate outcome indicators. The HAMD series of scales have good reliability and validity in the diagnosis of DD, and can be used to evaluate the severity and functional impact of the disease (71). Furthermore, the included studies revealed a significant correlation between HAMD scores and neuroimaging findings in the majority of patients with DD. These findings were observed in various brain regions, including the insula, precentral gyrus, cingulate cortex, precuneus, prefrontal cortex, and middle frontal gyrus. These regions are primarily situated in neural circuits such as the DMN, central executive network (CEN), and reward network. These correlations suggest that acupuncture treatment may regulate brain regions associated with emotional regulation, providing evidence for its therapeutic effects. Additionally, increased functional connectivity in regions related to emotional regulation may help clinicians identify biomarkers that predict treatment response, which could guide personalized treatment plans and enhance the effectiveness of acupuncture in treating depression. Finally, by understanding the underlying neuromechanisms of acupuncture effects, clinicians may be better equipped to use acupuncture as an adjunctive treatment for patients with depression, especially those who are resistant to conventional therapies.

In the imaging techniques mentioned, all of the studies included employed fMRI to reveal the brain activity of depression patients following acupuncture treatment. This preference is likely due to the numerous benefits of fMRI, such as being non-invasive, free from radiation, offering high spatial resolution, and its ability to perform both functional and morphological imaging simultaneously (33). Regarding analytical approaches, the majority of studies utilized ALFF and/or FC. ALFF is a method that reflects spontaneous neural activity at the voxel level and provides insight into the level of intrinsic brain activity during rest (72). FC, based on predefined regions of interest (ROIs) in resting-state fMRI, extracts the BOLD signal time series for each voxel within the ROI and computes the correlation with time series from other brain areas (73). Studies have indicated that combining ALFF and FC offers a more thorough understanding of the central mechanisms underlying acupuncture’s effects in treating depression (37, 74), and it is hoped that future research will focus on this.

### 4.2 Neuroimaging changes following the treatment of DD with acupuncture

#### 4.2.1 Functional neural abnormalities associated with DD

12 of the reviewed studies reported that patients with DD exhibited altered neural function when compared with HCs. The variations in ReHo, FC, and ALFF were primarily observed across different networks of the brain, including the DMN, CEN, and limbic systems. Brain regions such as the precuneus, amygdala, cingulate gyrus, precentral gyrus, superior frontal gyrus, pallidum, putamen, caudate nucleus, prefrontal cortex, and hippocampus were all found to be affected by DD. These findings agree with previous reports that the most significant neural differences between patients with DD and HCs are mainly distributed in the DMN and limbic system (43, 75). These neuroimaging results may inform potential targets for future studies exploring acupuncture-induced brain alterations in patients with DD.

#### 4.2.2 Acupuncture-related neural changes in patients with DD

Acupuncture-induced changes in the brain regions of individuals with DD were primarily observed in the cingulate gyrus, precuneus, insula, prefrontal lobe, middle frontal gyrus, cerebellum, putamen, angular gyrus, superior frontal gyrus, lingual gyrus, precentral gyrus, postcentral gyrus, thalamus, parahippocampal gyrus, amygdala, and caudate nucleus. Its main neuroimaging results are as shown in the **Table 3**.

The cingulate gyrus and amygdala belong to the limbic system and are heavily involved in emotional processing, learning, and memory (76). The precuneus plays an important role in the processing of self-related information and situational memory (77). The insula is responsible for the normal coordination of the cognitive and emotional systems and has been implicated in the onset of DD (78). The dorsolateral prefrontal cortex includes the superior frontal and middle frontal gyri, participates in cognitive and control functions, and can contributes to emotional regulation (79–81). The cerebellum is thought to be related to emotional experience, as abnormal cerebellar function can impair the ability to experience positive emotions (82). The putamen and caudate nucleus are important components of the corpus striatum, which functions as the core structure of the reward network. Abnormal corpus striatal function can impair neural reward mechanisms in patients with DD (83). Highly sensitive to stress and a key structure for emotional regulation, the hippocampus is associated with the onset of DD (84). The angular gyrus is responsible for the semantic processing, digital processing, attention, and memory of the human body (85). The thalamus is a relay station for various kinds of sensory information and participates in coordinating memory, emotions, and language (86). The parahippocampal gyrus forms part of a pathway that provides multiple types of perceptual information to the hippocampus. The abnormal functioning of either the parahippocampal gyrus or the hippocampus itself could affect various cognitive functions and thus cause or aggravate negative emotions (87, 88). Functional abnormalities in the precentral and postcentral gyri may induce somatic and depressive symptoms in patients with DD (89).

**Figure 2:**
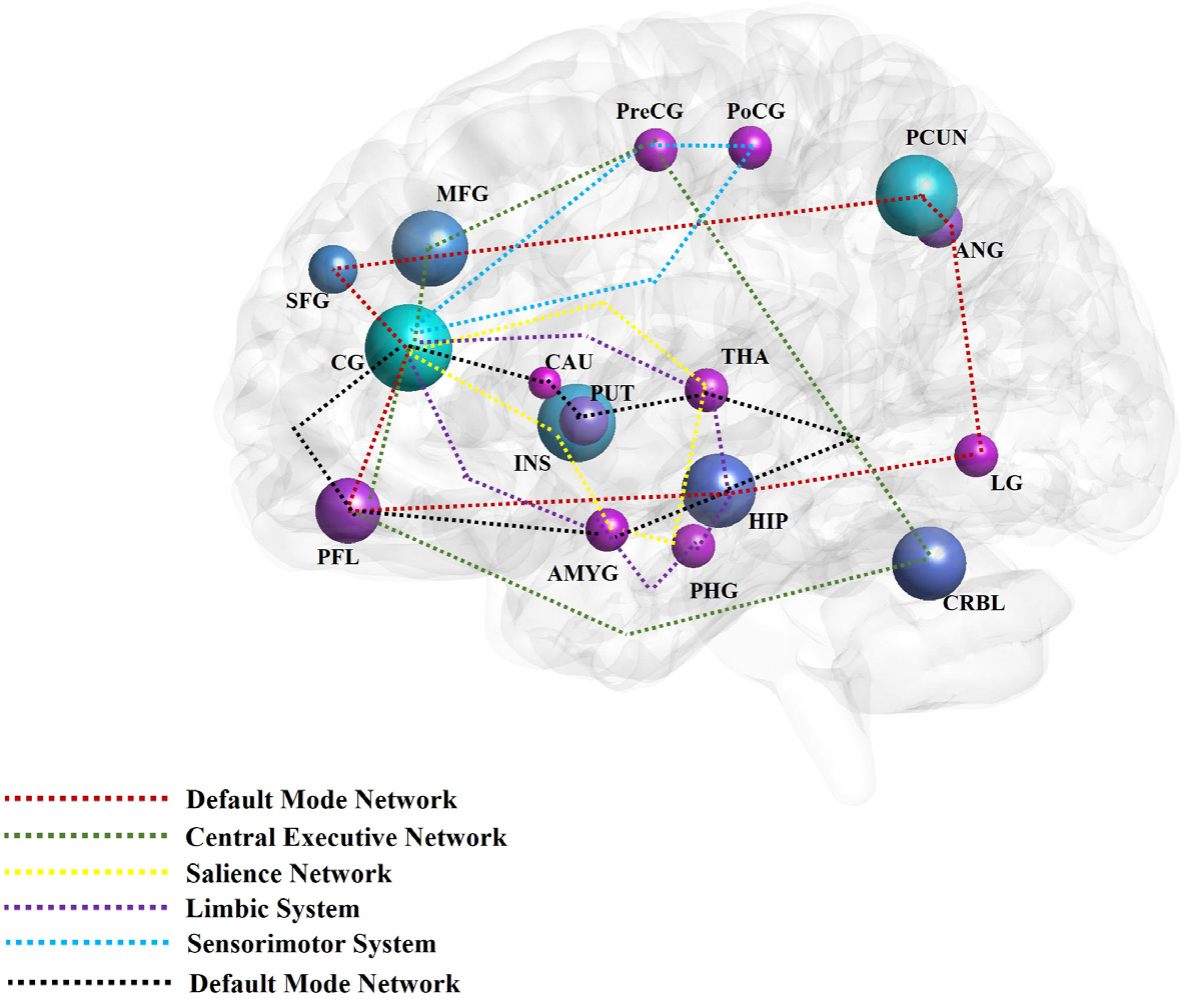
The main findings of acupuncture for depressive disorder by neuroimaging techniques. The high frequent reported brain regions that have been affected by acupuncture for depressive disorder in the included studies were noted with different colors. The different color circuits represent the different pathways or networks. CG, cingulate gyrus; PCUN, precuneus; INS, insula; MFG, middle frontal gyrus; PFL, prefrontal lobe; CRBL, cerebellum; HIP, hippocampus; PUT, putamen; ANG, angular gyrus; SFG, superior frontal gyrus; LG, lingual gyrus; PreCG, precentral gyrus; PoCG, postcentral gyrus; THA, thalamus; PHG, parahippocampus gyrus; AMYG, amygdala; CRBL, cerebellum; CAU, caudate nucleus; MOG, middle occipital gyrus; ORBf, orbitofrontal cortex.

The aforementioned brain regions are mainly distributed across the DMN, limbic system, emotional and cognitive regulation network, and reward network. Brain networks such as the CEN, SN, and sensorimotor network (SMN) are also involved in mediating the effects of acupuncture on patients with DD (**Figure 2**). Comprised of the prefrontal cortex, cingulate gyrus, precuneus, angular gyrus, superior frontal gyrus, and hippocampus, the DMN is responsible for generating and processing introspective thoughts and participates in emotional regulation (90, 91). The emotion regulation and cognitive networks consist of the limbic system, prefrontal cortex, corpus striatum, and thalamus, as well as their connections to the amygdala, hippocampus, prefrontal cortex, caudate nucleus, cingulate gyrus, thalamus, and other brain regions (92). The reward network plays an important role in the pathogenesis of DD (93). Though the cortical-basal ganglia circuit functions as the core of the reward network, the cingulate cortex, prefrontal cortex, and corpus striatum are key structures in the network, and their interaction with the amygdala, hippocampus, and thalamus is essential to regulating the reward network (83). The SN includes the cingulate cortex, insular lobe, and amygdala; all these regions feature strong connections to the limbic system. The CEN is comprised of the prefrontal cortex, cingulate cortex, and precentral gyrus among other brain regions. Both the SN and CEN play important roles in emotion and cognition (94). Composed of the postcentral gyrus, precentral gyrus, and cingulate gyrus, the SMN plays an important role in the onset of somatic and depressive symptoms associated with DD.

In summary, the alterations in brain activity observed in individuals with DD following acupuncture treatment are marked by collaborative disruptions across various brain networks and regions. Subsequent investigations into the association between acupuncture and DD ought to prioritize elucidating the fundamental mechanisms through which acupuncture may mitigate DD symptoms. These inquiries should emphasize a thorough examination of the aforementioned brain regions and networks, as well as their interconnections. For example, studies have shown that acupuncture can significantly regulate the activity and connectivity of extensive neural networks to alleviate depressive symptoms. This is manifested by a direct or indirect reduction in the connectivity between the posterior DMN and the emotional and reward networks, while enhancing the connections between the anterior and posterior DMN, the anterior DMN and the CEN, and the CEN and the emotional and reward circuits (29).

### 4.3 Strengths of the present review

Prior systematic reviews have primarily concentrated on assessing the effectiveness and safety of acupuncture as a treatment for DD. Although a systematic review of magnetic resonance imaging studies on acupuncture therapy in depression has been published (95). However, compared to this review, the above study, 1) The search was limited to four pertinent electronic databases, potentially resulting in incomplete data retrieval and the exclusion of some eligible research. 2) The data retrieval timeframe extends until October 20, 2020, with three years having elapsed since that date. It is conceivable that recent neuroimaging studies on the efficacy of acupuncture treatment for DD have not been adequately considered. 3) Acupuncture intervention measures such as taVNS and tVNS are excluded, and incomplete intervention details may also lead to incomplete retrieval. 4) Moreover, it only focuses on the changes of some brain regions’ functions caused by acupuncture, but does not summarize the relevant brain networks, which cannot intuitively present the central mechanism of acupuncture’s effect on depression.

In order to offer a comprehensive and up-to-date examination of the topic, this review will redirect its attention to neuroimaging studies on the efficacy of acupuncture in the treatment of DD. Additionally, this study will provide a detailed analysis of the neural activity alterations induced by acupuncture in individuals with DD. Moreover, it considers the association between these findings and clinical parameters with the aim of establishing a framework for future investigations into the neural mechanisms underlying a specific acupuncture technique for treating DD.

### 4.4 Limitations

Although this study is the latest and most comprehensive neuroimaging review on acupuncture treatment of DD, the following limitations still exist. Firstly, differences were found between the included studies in their methods of intervention: specifically, in their needle retaining time, intensity, and frequency. As a further example, while most studies discussed the VNS- and EA-induced brain changes in patients with DD, the waveform was mainly administered as a density wave with a frequency and intensity of 4–5/20 Hz and 4–8 mA, respectively. Differences in the parameter settings of electroacupuncture (EA) were noted, with the waveform predominantly administered as a continuous wave at frequencies of 1–2 Hz and intensities of 2–3 mA. These variations may have influenced the outcomes of neuroimaging studies. Furthermore, the utilization of MRI technology, including resting-state functional MRI (rs-fMRI) and functional MRI (fMRI), in the included studies lacked integration across multiple studies, resulting in a reliance on a single imaging modality. Furthermore, the included studies limited their inquiry to single or several independent brain regions or networks related to the effect of acupuncture on patients with DD. The lack of an integrated analysis of brain regions or networks further compromised the identification of a mechanism underlying the treatment’s therapeutic effect. Thirdly, the reviewed studies included far more female patients with DD than male patients accounted. Although the literature suggests that female DD patients are more common than male DD patients due to reproductive events elevating the risk of DD, comparing male and female DD patients in the same study would enhance heterogeneity of the study’s findings. In addition, many of the studies included in the review had relatively small sample sizes, which could reduce the statistical power of the findings and affect the generalizability of the results. Larger sample sizes and more high-quality RCTs are needed to strengthen the evidence for acupuncture treatment of depression. Finally, as the 26 included studies were performed in mainland China, their neuroimaging findings have limited applicability to countries and regions outside China, and their reliability as a reference is thus diminished.

### 4.5 Outlook

The literature would benefit from neuroimaging studies on the use of acupuncture in treating DD performed outside of China in the future. Such research should strictly follow the Cochrane Handbook, feature superior RCT design, employ a rigorous randomization process, adequately handle dropout data, and ensure excellent methodological quality. It is imperative for future research on the effects of acupuncture on patients with DD to incorporate advanced imaging techniques and analytical methods in order to enhance understanding of the treatment’s mechanisms of action. A multimodal approach should be adopted to investigate the interactions among various brain regions or networks, with equal emphasis on assessing alterations in neural function and structure. Additionally, in order to minimize the influence of bias resulting from small sample sizes, it is advisable to include participant populations of maximal size. The need for a large sample should be balanced against the careful consideration of recruiting patients with homogenous characteristics, including gender classification, to facilitate the exploration of neural mechanisms underlying gender-based differences in acupuncture treatment for DD. In addition, future studies should strengthen longitudinal designs to assess the durability of treatment effects and explore how acupuncture influences brain activity over a longer period, which may provide insights into the mechanisms for sustained benefits. Ultimately, it is desirable to achieve standardization and uniformity in the method of administering acupuncture, such as the parameter settings for VNS and EA, to minimize bias resulting from differences in intervention methods.

## 5 Conclusion

This review provides evidence that acupuncture induces changes in brain function in distinct neural regions among individuals with Depressive Disorder, encompassing the Default Mode Network (DMN), limbic system, emotional and cognitive regulation network, reward network, Central Executive Network (CEN), Salience Network (SN), and Sensorimotor Network (SMN). Notably, these changes were primarily observed in the cingulate gyrus, precuneus, insula, prefrontal lobe, amygdala, superior frontal gyrus, cerebellum, putamen, caudate nucleus, middle frontal gyrus, hippocampus, angular gyrus, thalamus, parahippocampal gyrus, precentral gyrus, and postcentral gyrus. However, the studies included in this analysis only used MRI neuroimaging technology. Hence, additional randomized controlled trials incorporating high-quality, multimodal designs and enhanced integration of neuroimaging data are necessary to further elucidate the underlying mechanism of acupuncture in the treatment of DD.

## Data Availability

The original contributions presented in the study are included in the article/supplementary material. Further inquiries can be directed to the corresponding authors.
